# Implementation of a workplace smoking ban in bars: The limits of local discretion

**DOI:** 10.1186/1471-2458-8-402

**Published:** 2008-12-08

**Authors:** Theresa Montini, Lisa A Bero

**Affiliations:** 1 New York University, 423 East 23rd Street, VET-16N, New York, NY 10010, USA; 2 School of Pharmacy, 3333 California Street, Box 0613, University of California, San Francisco, San Francisco, CA 94143-0613, USA

## Abstract

**Background:**

In January 1998, the California state legislature extended a workplace smoking ban to bars. The purpose of this study was to explore the conditions that facilitate or hinder compliance with a smoking ban in bars.

**Methods:**

We studied the implementation of the smoking ban in bars by interviewing three sets of policy participants: bar employers responsible for complying with the law; local government officials responsible for enforcing the law; and tobacco control activists who facilitated implementation. We transcribed the interviews and did a qualitative analysis of the text.

**Results:**

The conditions that facilitated bar owners' compliance with a smoking ban in bars included: if the cost to comply was minimal; if the bars with which they were in competition were in compliance with the smoking ban; and if there was authoritative, consistent, coordinated, and uniform enforcement. Conversely, the conditions that hindered compliance included: if the law had minimal sanctions; if competing bars in the area allowed smoking; and if enforcement was delayed or inadequate.

**Conclusion:**

Many local enforcers wished to forfeit their local discretion and believed the workplace smoking ban in bars would be best implemented by a state agency. The potential implication of this study is that, given the complex nature of local politics, smoking bans in bars are best implemented at a broader provincial or national level.

## Background

In the last decade, enacting workplace smoking bans in bars has become common. While the state of California was the first to prohibit smoking in bars in 1998, subsequently 16 nations (Bermuda, Bhutan, British Virgin Islands, Commonwealth of Puerto Rico, Czech Republic, England, France, Ireland, Italy, New Zealand, Northern Ireland, Norway, Scotland, Sweden, Uruguay, and Wales), six of the eight Australian states and territories, 12 of the 13 Canadian provinces and territories,[1] and 19 of 50 United States of America states have outlawed smoking in bars. [2]

While passing legislation takes a great deal of activist coordination and political consensus building, the law actually has to be implemented if workers are to benefit from it. We present findings from our case study of the implementation of the smoking ban in bars in the state of California, from the initial presumed voluntary compliance with the law, through the later stages of enforcement of the law. We studied the policy process as it evolved, with the goal of elucidating the conditions that facilitate compliance with the smoking ban in bars, and the potential barriers that hinder compliance. Our analysis follows the trajectory of recent examinations of policy that conceptualize implementation as the "process of change that occurs after the adoption of a policy and before the routinization of operations, activities, or tasks that are governed by the policy."[3] California Labor Code 6404.5 states that, "No employer shall knowingly or intentionally permit, and no other person shall engage in, the smoking of tobacco products in an enclosed space at a place of employment" (Additional file 1). If there is smoking in a workplace, employees or members of the public may confidentially report violations to local law enforcement agencies. In terms of penalties, there is a series of fines: first violation $100; second violation (within one year) $200; third and subsequent violations (within one year) $500. The law also allows for the State of California Occupational Safety and Health Administration (Cal OSHA) to become involved, and the state can levy up to $70,000 in fines. There is also the possibility of violators to be subject to unfair business practice lawsuits from their competitors who comply with the law.

When this law took effect in 1995, it required that all workplaces except bars be smoke free. Most employers voluntarily complied, but in 1998 when the smoking ban was extended to bars, generally stand-alone bars did not comply immediately or voluntarily. [4-7] Thus, the extension of the smoking ban to bars is a case example of an implementation process that required both start up and fine-tuning.

Assemblyman Terry Friedman introduced smoke-free workplace legislation in the California State Assembly in December 1992. According to Friedman's commentary at various legislative committee meetings, his major impetus was the issue of workers' compensation. [8,9] The legislation was introduced following a California workers' compensation insurance fund award of $85,000 to a restaurant waiter/manager who had suffered a heart attack, [10] having no risk factors other than exposure to tobacco smoke in the restaurant. The plaintiff's case invoked the 1986 *US Surgeon General's Report *delineating the health consequences of involuntary smoking for healthy nonsmokers,[11] which included cancer and heart disease. After this 1990 court case, but before the legislation was passed in 1994, there were two additional scientific reports on the hazards of exposure to tobacco smoke that could also be used by workers in lawsuits against employers who failed to provide a safe smoke-free workplace: the 1991 National Institute for Occupational Safety and Health reaffirmed the health consequences of passive smoking and recommended that non-smoking workers be protected from involuntary workplace exposure to tobacco smoke;[12] and the 1992 federal Environmental Protection Agency established that tobacco smoke was a Group A (known human) carcinogen[13] with no safe exposure levels.

Any effort to reduce public exposure to tobacco smoke typically draws the support of public health activists. While the legislation was being considered, it was referred to as Assembly Bill 13 or AB13. During the legislature's debate of Assembly Bill 13, public health activists lobbied for local control and enforcement. [8,9,14] At the time of the debate, many municipalities in California had strong smoke free ordinances. Activists wanted to assure that a statewide law would not pre-empt stronger local ordinances or regulations, and believed that local control would assure the maintenance of local norms.

During a 1994 Judiciary Committee hearing, Friedman stated his intentions to have the enforcement of the law handled at the local level rather than at the state level:

We leave it to local government to decide how it should be enforced. It's entirely within the control and the flexibility and discretion of local government to decide how to enforce it. I don't think that this is the time to impose a mandate, to impose those costs on local government and to force them to set up a bureaucracy. [15]

The legislation that passed simply stated that local elected officials were to designate a local government entity as enforcer. On one hand, this vague enforcement provision could be seen as the inevitable outcome of the legislature grappling with a politically fraught issue. As Brodkin has observed,

Ambiguous policy is produced when politicians seek to avoid thorny political issues and, effectively, "pass the buck" to the bureaucracy. This strategy (whether consciously intended or not) enables politicians to claim credit for grand policy objectives while reserving the opportunity to blame the bureaucracy later for political interpretations that generate political heat. [16]

The legislation was ambiguous because it did not specify who was to serve in what implementation role, primarily because local governments do not typically have local labor law enforcement apparatus. Policy analysts working from a top down perspective[17] would predict that because this law involved many and varied organizations to implement it, implementation would be uncertain and difficult.

On the other hand, as Ingram and Schneider have observed,

From a grass roots perspective, vague and nonspecific statutes may actually be virtues, not just because clarity is impossible to achieve, but also because ambiguity provides maximum leeway to local level implementers permitting them to adapt the statute to local needs. [18]

Policy analysts refer to the above-mentioned "maximum leeway" as local enforcer discretion. Policy analysts working from a bottom up perspective[19] would predict that the greater the complexity anticipated in the varied workplaces, especially numerous small, independently owned workplaces such as bars, the greater the discretion that enforcement officials would need to implement the policy.

We studied this particular implementation effort by tracking the actors involved, their decisions, and the consequences of such. This state law prohibiting smoking in workplaces can be characterized as a top-down initiative with local groups and individuals charged with its implementation. We followed Brodkin's[16] suggestion that implementation be analyzed as policy process, and investigated why, when, and how this policy was defined and redefined in the field.

## Methods

We collected interview data from three groups: bar employers, local enforcement officials, and activists who supported implementation. We conducted the interviews from mid-1998 to 2000. We anticipated that our response rate could be affected by bar owners' reluctance to put themselves in a situation where they would admit breaking the law, and local enforcers reluctance to put themselves in a situation where they would admit failing to enforce a law, therefore we secured a *Certificate of Confidentiality *issued by the National Institutes of Health to protect participants in this research study from any breach of confidentiality. Upon inviting each subject to participate in this research study, we informed them that the confidentiality of any information they would give us was protected legally by the *Certificate of Confidentiality *and ethically by the University of California-San Francisco Committee on Human Research (H2758-14852-03).

### Bar Employers

We sampled bar employers (owners) from the entire state, defining our population as all California bars, taverns, and gaming clubs (henceforth referred to as "bars"). The California Department of Alcoholic Beverage Control (ABC) issues liquor licenses to all bars, and we included in our sample the types of licenses that are issued to "stand alone" bars, that is, bars that do not serve meals:

Type 42: On-sale beer and wine public premises (N = 1,333)

Type 48: On-sale general public premises (N = 3,503)

Type 61: On-sale beer public premises (N = 36)

This set of licenses resulted in a population of 4872 bars. We stratified the sample by county (N = 57; 58 California counties minus one county with no stand-alone bars) and randomly sampled 5% of liquor licenses from each county. We used random sampling to reduce selection bias. We sent letters to the bar owners listed on the license to the address of the bar, and a follow-up letter one month later. In the letter we requested a confidential interview with the bar employer about their experiences with the smoking ban in bars, either in-person or by phone. We sent out 231 letters requesting an interview, and 57 bar owners responded. Of those that responded, 28 agreed to be interviewed, 20 refused to be interviewed, and nine bar owners told us that they had sold their business.

In order to assure that the bar owners who agreed to be interviewed were not operating bars that were different from those that refused to be interviewed or did not respond, we compared three groups (those who agreed to be interviewed, those who refused to be interviewed, and those who did not respond at all) on four economic census data indicators[20] that were based on the geographic area of the bar's postal code:

1) The number of bars in their postal zone – to assure that our respondents were not coming from areas where they had either more or less competition.

2) The median sales receipts of bars in their postal zone – to assure that our respondents were not coming from an area where the businesses were either more or less prosperous.

3) The median annual payroll of bars in their postal zone – to assure that our respondents were not coming from an area where employees earned either more or less.

4) The median number of paid employees in their postal zone – to assure that our respondents were not coming from a geographical area that had either more or fewer paid employees.

Table 1 lists summary economic indicators for the three groups of bar owner respondents.

**Table 1:** Economic indicators of bars in sample

**Bar owner response group**	**Average number of bars in postal zipcode**	**Median sales or receipts**	**Median annual payroll**	**Median number of paid employees**
Agreed to be interviewed (N = 28)	67	25,000,000 to 49,999,000	5,000,000 to 9,999,000	500 to 999
Refused to be interviewed (N = 20)	68	10,000,000 to 24,999,000	5,000,000 to 9,999,000	500 to 999
Did not respond	62	25,000,000 to 49,999,000	5,000,000 to 9,999,000	500 to 999

The results of the *Kruskal Wallis *test indicated that there was a statistically significant difference (p = 0.045) between the bar owners who refused to be interviewed and the other two groups in that the bar owners who refused to be interviewed operated bars in postal zones in which the median sales receipts were lower.

Of the bar owners who agreed to be in the study, we were able to contact and interview 28 either by phone or at their bar. The in-depth semi-structured interviews of bar owners were guided by the interview schedule in Additional file 2.

### Local Enforcement Officials

Our goal was to interview at least one enforcement official from each of the 57 California counties that had stand-alone bars. Soon after we began this study, a state-funded organization compiled and published a list of designated enforcement personnel by county. [21] We sent a letter of invitation to two randomly selected enforcement officials from each of the 57 California counties that had freestanding bars, and a month later sent a follow-up. In the letter we asked the enforcer for a confidential interview, either in-person or by telephone, about their experiences with the smoking ban in bars. Forty-five local enforcement officials responded: 30 agreed to be interviewed, and 15 declined to be interviewed.

In order to assure that the local enforcement officials who agreed to be interviewed were not working in counties that were different from those that refused to be interviewed or did not respond, we compared three groups (those who agreed to be interviewed, those who refused to be interviewed, and those who did not respond at all) on four economic census data indicators[20] that were based on the geographic area of the county:

1) The number of bars in their county – to assure that our respondents were not coming from areas where they had either more or less bars to monitor.

2) The median sales receipts of bars in their county – to assure that our respondents were not coming from a county where the businesses were either more or less prosperous.

3) The median annual payroll of bars in their county – to assure that our respondents were not coming from a county where employees earned either more or less.

4) The median number of paid employees in their county – to assure that our respondents were not coming from a county that had either more or fewer paid employees.

Table 2 is a summary of economic indicators for each of the three groups of law enforcement respondents.

**Table 2:** Economic indicators of counties of law enforcers in sample

**Local enforcer response group**	**Average number of bars in county**	**Mean county sales or receipts**	**Mean county average payroll**	**Mean county number of employees**
Agreed to be interviewed (N = 30)	742	383,193,000	105,156,000	10,865
Refused to be interviewed (N = 15)	1,120	649,998,000	176,856,000	18,462
Did not respond (N = 12)	1,327	770,775,000	205,007,000	20,387

The results of the *Kruskal Wallis *test indicated that there were no statistically significant differences (p > 0.35) in the economic indicators of the counties of the local enforcement officials who agreed to be interviewed, those who refused to be interviewed, and those who did not respond. Of those who agreed to be in the study, we were able to contact and interview all 30. The in-depth semi-structured interviews of local enforcement officials were guided by the interview schedule in Additional file 3.

### Activists

We intended to interview both activists who worked for or against the implementation of California Labor Code 6404.5. We identified activists by doing a *NEXIS*^® ^online search of newspapers, magazines, and broadcast transcripts dated December 7, 1992 to January 19, 1999 using the terms: "California" and "Assembly Bill 13" or "AB13" or "AB 13." Our search produced 138 news articles, 37 magazine articles, and transcripts from four broadcasts. We reviewed these news accounts, and recorded the name and affiliation of any activist, supporting or opposing the workplace smoking ban, who was mentioned in the news source. We were able to find addresses of 46 activists who had supported Assembly Bill 13, and 11 activists against Assembly Bill 13. We sent each a letter that explained how we knew of their activism (full citations of news sources were given), and requested an interview, either in-person or by telephone, about their advocacy for or against the smoking ban in bars. We sent a follow-up letter a month later. Of the 46 activists who supported the smoking ban in bars, 12 agreed to be interviewed, two declined to be interviewed, and 32 did not respond. Of the 11 activists against Assembly Bill 13, two declined to be interviewed, and nine did not respond. Therefore, we interviewed only activists who were in favor of Assembly Bill 13 (N = 12) guided by the interview schedule in Additional file 4.

### Data Analysis

Interviews lasted an hour to an hour and a half, and interviews were audio taped (18 bar owners, 19 local enforcers, and 12 activists) unless the respondent would not allow recording, in which case we asked for permission to take handwritten notes (10 bar owners, 11 local enforcers). The audiotapes were transcribed *verbatim *and we analyzed the transcripts using grounded theory procedures. [22-25] When doing open coding, we labelled the ideas contained in passages of text. For example, when coding a transcript of an interview of a bar owner some concepts that were identified included "wanting to comply," "finding loopholes," "feigning compliance," and so forth. We grouped these concepts into a larger category of "compliance." Certain characteristics or properties, such as "degree of compliance" or "modifying compliance" described the category "compliance." Dimensions for the properties of "compliance" ranged from "complete compliance" to "non-compliance."

Then we integrated data and codes across transcripts and respondents, and from the data generated hypothesized relationships to explain and clarify categories. Continuing the example above, we tracked a relationship between "non-compliance" and the perception of an "uneven playing field." Therefore, we were able to link categories, hypothesize relationships, and re-examine the data to verify our analysis.

Finally, we followed concepts and categories in light of changing situations or circumstances. Continuing with the example of "compliance," we considered how respondents altered strategies over time in response to changing situations. For example, we tracked alterations along the dimension of "non-compliance" to "compliance" as bar owners responded to changing conditions of a "level playing field" or the initiation of "enforcement."

## Results

We begin by presenting the issues central to the bar owners because in January 1998, when few enforcement mechanisms were being put into place, the onus was on the bar owners to comply voluntarily. Given that the enforcement effort came later, we next present issues regarding enforcement. Finally, we close with findings from the interviews of tobacco control activists, who responded to events as they unfolded.

### Bar Employers

In general, bar employers feared that their compliance with the workplace safety law would threaten their sales revenues. Therefore, they complied with the law to the degree they believed they could afford to. When interviewed, bar employers reported that they were in compliance if they had made any effort to comply. However, when asked to elaborate, we learned that compliance was an elastic concept, and heard them describe a gradation ranging from non-compliance through various degrees of compliance.

A bar owner was likely to be in compliance if the costs of compliance were minimal, for example, if the bar had an existing accessible, safe, and comfortable (usually concerning weather) area where patrons could smoke outdoors. This bar owner explains the ease of complying given that the bar was located in a temperate coastal region and already had an outdoor patio:

We have two separate bars, and then we have one very large outdoor patio space. Just about anybody that had the patio spaces – I think those bars actually picked up a little bit of business because people could smoke outside. I have friends that don't smoke – those people would consciously not go to a bar where they knew there was going to be a lot of smoking. We probably had a return of those people.

Most bar owners believed that the smoking ban would reduce profit, so they were caught between wanting to abide by the law, yet fearing the consequences of full compliance. They engaged in modified compliance, complying when the consequences were negligible, by allowing smoking at some times (*e.g*., after 5 p.m.), on some days (*e.g*., during special events), or in some areas, as does this bar owner:

We've quite a big bar, so what we've done is we've allowed it in the poolroom only, nowhere near the food.

Later in the interview, this particular bar owner explained that the bar had a ventilation hood over the range, and continued, "So, I've explained to them, 'Girls, you're in no danger. Look how much this hood takes away.' You know, I mean I'm not putting them in danger. They agreed to take the job back when every bar was allowing smoking." Many of the bar owners were unaware of the scientific research available at the time regarding the inability of ventilation to remove environmental tobacco smoke. [26-28]

Other bar owners looked for loopholes in the law. For example, this bar owner understood that the law was an employee protection measure and deduced that if they had no employees the law would not apply to them:

Two ex-employees and I started the bar in (city): the three of us went down there and opened this place, and the three of us worked it. So, when the law came into effect we did not have to abide by it, we had an exemption because we had no employees.

From a business standpoint, the sanctions to penalize non-compliance were minimal. Yet, some bar owners complied because they feared that the attention generated by a smoking violation could lead enforcement authorities to notice other, far more consequential, violations. This was usually the case if the bar owner was under surveillance because of prior violations that threatened their liquor license, for example, underage drinking, exceeding maximum capacity, or drug trafficking. In these cases the bar owner generalized compliance to all legislation or regulations, including the smoking ban. An owner of both a bar and an adult club explained the decision to comply with the smoking ban:

Primarily with the adult clubs, I am very tightly monitored by the police, in the extreme. And, it just doesn't make sense for me to allow anything that's illegal because the police will use that as a means to harass me. So, I've been very strict about enforcing the no smoking policies.

We found that bar owners were very concerned with issues of equity: that all businesses in their area were subject to the same enforcement. If other bars in their area were complying, then the bar under study was likely to be in compliance. If other bars in the area allowed smoking, bar owners feared losing their customers, and therefore were less likely to be in compliance. If other bars in the area allowed smoking, even if the bar owner wanted to comply, the probability of sustaining initial compliance was low, as can be heard in this interview:

The law was blatantly ignored by most – I'd say 90% of the bars. There's a bar two doors down from me that has always been kind of a hip dive and all my smokers went there. I would come down on a Friday night and I would have twelve people, and the bar next door was packed. That went on for a long time with me contacting the police and saying, "What's going on? There's a law going on here! I mean I understand civil disobedience, if someone wants to break the law, they should be able to break it. But shouldn't they be the ones that are being penalized economically, not someone who's obeying the law?" After a few months of, I mean, serious, serious, economic disadvantage, my employees were just ready to quit or take jobs at other bars, people that had worked for me for five or six years. I did a compromise where I allowed people to smoke after 10 o'clock at their own risk. I wasn't in a position to be able to enforce that law and to march boldly toward economic ruin.

As per this owner, over time, without adequate enforcement, even bar owners who had initially complied with the law retreated from compliance because they feared the financial consequences of operating a business on an uneven playing field.

We were not able to determine whether bar employers understood or believed the seriousness of the health consequences of exposure to tobacco smoke. However, complying with a workplace smoking ban was a particularly onerous task given that bar owners/employers were structurally compromised: the business has to attract customers to make a profit; and those very customers were producing the toxin unconnected to the service delivered. If an employer has to protect workers from exposure to asbestos or radon, they can have the substance removed from the workplace, or they can move the workplace to a toxic free location. If an employer has to protect workers from exposure to by-products of a manufacturing process, such as arsenic or benzene, they can change the production process or they can find safer substitutes. While an employer has control over where the business is located, how employees work, and how manufacturing is conducted, protecting workers from tobacco smoke was different in that those in the service industry have little control over customers' behavior, and in fact, controlling customers is often counter-productive to the goals of making a profit. The bar owners understood that compliance with this particular code involved the cooperation of their customers, as this bar owner explains:

The health department – they are a routine investigative agency, and they come in periodically and go around and check everything they are supposed to. If there are any violations, we correct them; it is just a normal process. Smoking has become a thing that the customers get involved with; and then, you know, they become belligerent.

Ultimately, the bar employers' lack of compliance with the law can be attributed to their reluctance to ask customers to stop smoking: they were unwilling to engage in efforts to change their customers' behavior in order to make their employees' workplace safer. Their compromised situation made outside enforcement efforts more important.

### Enforcement

The law specified that local elected officials were to delegate the enforcement of Labor Code 6404.5 to a local government entity. Because there had been widespread voluntary compliance to Labor Code 6404.5 before its extension to bars in 1998, local officials had little enforcement experience, as this county health worker explained:

Mainly what we found before bars enforcement was that letters, phone calls, and an occasional site visit took care of the problem. With the bars, nothing could have been further from the truth. We found that we needed to go out to the law enforcement agents in these targeted areas where we were having problems and ask them if they would be willing to actively enforce these laws and partner with us to make sure there was a uniform application of them.

The local government entity's enforcement experience varied. When dealing with tobacco, health department officials tended to have more experience with health education campaigns. A city health department director explained, "Typically we don't enforce, we don't protect workers. We protect the public." On the other hand, a police chief was much more comfortable with enforcement:

You have a person committing a crime, whether it be selling heroin or smoking a cigarette. When it comes down to the nuts and bolts of how do you get the person not to do it, not commit the crime anymore, it's basic law enforcement.

We consistently heard about how long it took localities to begin enforcing, as this enforcer relayed:

I guess there had been some previous meetings, but it has been about two years now, and we in (county) have taken minimal enforcement action. So, they were kind of saying, "OK, look, the warning period is over. We are gonna get serious about it." So, we had the big meeting, and then we developed a game plan in our city.

In some areas, enforcement was delayed as the "hot potato" of enforcement responsibility was passed from one local agency to another. In response to a question about the pros and cons of situating enforcement at the local level, this building code inspector replied:

I think that it was a definite disadvantage. It made it almost twice as hard, probably because one little agency thought they could push it off on the other agency. The local police department said, "It's the health department's job." The health department said, "It is the fire department's job." It just went around in a big circle. It was almost two years before we just finally said that's enough; this is what's gonna happen.

The bar owners were aware that the responsibility for enforcement was passing from entity to entity, as this respondent relayed:

TM: Are you saying that right now the city building code inspectors are responsible for enforcing the law?

**Bar Owner**: Right.

TM: Do you know why?

**Bar Owner**: Probably because the health department was too busy. Everybody seems to be putting it off on everybody else, it's like nobody wants to deal with it. I mean, you know, these enforcement people don't really want to do it.

Berman reminds us that policy is "implemented by program operators who may or may not be in sympathy with the plans, may or may not have even understood them, but in any case will certainly be governed by their own motives and imperatives, both personal and programmatic."[17] Among various political entities in a given locale, different actors held different views on the importance of enforcing smoking restrictions. For example, often health department officers would cite bar employers only to find their work undone when the district attorneys failed to press charges, or the regional judge dismissed the charges. Bar owners noticed the lack of consistency, as indicated in this account:

I went to court and the district attorney said, "Well, let's go outside and talk." He said, "I smoke. I think it's a bad law too, but I have to enforce it. What they're trying to do is fine you $300 for the offense and be on probation one more time." So I said, "Well, we'll talk to the judge." And he said, "I'm going to recommend a $50 fine, and next time anybody's caught it will be a $200 fine." So I said, "Well, OK. That's better than $300."

This lack of coordination with, and support from, other enforcement entities was a central concern for frontline enforcers. One enforcer captured the essence of the issue: "And, the nice thing is that when I catch the bartender smoking, I can write them up for a *Health and Safety Code *violation, which is much easier to make stick."

Jacobson and Wasserman contend that most states that pass clean indoor air laws delegate enforcement responsibility to local agencies without designating additional funding. [29] California Labor Code 6404.5 was one such "unfunded mandate." Not having funds meant that locales were not able to designate personnel nor reassign staff time to implement and enforce, even in places where local government administrators were in favor of the smoking ban in bars. Local governments were further constrained by prohibitions against using tobacco tax revenues for anything but prevention and education. Consequently, tobacco control staff, those who had technical expertise in tobacco issues, were prohibited from engaging in enforcement because they received tobacco tax funds. Even when locales made a good faith effort to enforce, having to work within their funding parameters meant that they could only assign limited personnel to enforcement, and/or had to limit enforcement to regular working hours (9-to-5). Working within these confines demanded Herculean exertion, as described by this enforcer:

We have one person, me, working on 1,400 bars...The fear of citation has to be a little bit more than what it is now because I think they've figured out the numbers: in that you have one guy and 1,400 bars in the county, and he can't be in two communities at the same time. So, I think that people are banking on the fact that they are going to get away with it. It's like, if there was only one highway patrol for the whole county, what would the speeding be like? It would be out of control.

The most expensive enforcement activities were those in which two workers were needed. Sometimes this was because health department workers were physically threatened while inspecting bars, as this enforcer describes:

The police departments have been very helpful to accompany me in places that are kind of ugly. In fact, it's gotten to the point now where, with just about all of them, we have to have escort...I had somebody mail me a bullet the other day in an anonymous note...I had one guy say after I cited him when I was leaving, "Well, I just hope nothing happens to your car when you drive home," and made some vague sinister reference to my family.

Tobacco control enforcers having to take precautions to protect themselves from harm has also been reported by DiFranza *et al*. [30] who used "sting" operations in Massachusetts to deter merchants from selling tobacco to minors; and by Ashley *et al*. [31] who enforced smoking bans in Ontario high schools. While escorts are prudent given the strong feelings elicited by the smoking bans in bars, the coupling of workers cuts the workforce's productivity in half while doubling the salary liability.

Many of the enforcers we interviewed currently, or previously had, enforced other codes, for example, the health department's enforcement of no smoking when handling food or drink, the building code department's enforcement of the standards of the Americans with Disabilities Act, the fire department's enforcement of maximum occupancy standards, and the police department's enforcement of public drunkenness prohibitions. Therefore, the enforcement of the workplace smoking ban was folded into an ongoing relationship between bar owner and enforcer. When asked to compare the enforcement of the smoking ban in bars to other codes they enforced, local enforcers unanimously contended that enforcing the smoking ban in bars was more difficult because customers were causing the workplace safety hazard, as opposed to employers or employees.

Following a series of court cases in which bar owners challenged the enforcement of law, the local enforcers lost ground. This enforcer explained:

In our community, the district attorney would not allow us to cite the owner initially. We are required to cite the patrons to establish a pattern of non-compliance. Our district attorney, although I said has been very supportive over the years, read the law and said he felt he couldn't bring a case before a judge unless we had a couple of citations of patrons on different days that showed that there was more than a chance occurrence there was smoking taking place in the establishment.

Having the police cite patrons is a step toward the goal of the law in that it reduces the exposure of workers to tobacco smoke, and hence should reduce workers' compensation cases. However, police ticketing patrons does not necessarily meet the intent of the law, which specified that local elected officials should designate local enforcers who would assure that employers provided safe workplaces for their employees. When local officials cite employers for failing to provide a smoke-free work environment, it forces bar owners' involvement. The strategy of the police citing customers changes both who does the enforcement (police instead of designated local officials) and who must act to be in compliance (customers instead of employers), effectively letting the bar owners "off the hook." From the perspective of top down policy analysis, the policy of ticketing patrons could be viewed as a slipping away from the intended target of the labor law – employers. From the bottom up perspective, police ticketing patrons could be viewed as using local discretion to develop an innovative response to a problematic situation. The shift from focusing on employer compliance to the police ticketing patrons could be conceptualized as an adaptation to the difficult circumstances of not having enough local resources to provide the level of enforcement that was needed.

As did the enforcer quoted earlier, many enforcers often compared the workplace smoking ban in bars to speeding: that people in general speeded, and that the only way to deter speeding was to have 24/7 potential of substantial sanctions. The types of local government agency personnel assigned to enforcement usually worked 9-to-5 Monday to Friday, while the police worked around the clock. Enforcers knew that a prosperous bar owner could absorb increasing fines of $100/$200/$500 as the cost of doing business. However, an individual customer would be more strongly impacted by a series of personal citations.

From the local enforcers' perspective, they were losing ground. They made comparisons with other enforcement efforts that worked. They contended that enforcement would be more effective if it was handled at the state, rather than local, level of government, as per this enforcer:

If this was an enforceable citation by Cal OSHA, it could have been done in a month. People in bars and restaurants and any kind of industry, they shake in their boots when they get a Cal OSHA violation because the fines are so large and they have to go all the way to Sacramento for their hearings – it just makes it impossible for them.

The enforcers knew that there was one sanction that was extremely important to bar owners, that was their liquor license. Therefore, almost all enforcers interviewed saw the prohibition of smoking as a factor that should be evaluated by the state Department of Alcoholic Beverage Control (ABC) when considering whether to renew or revoke a bar liquor license. A local police chief who was actively enforcing the law in a rural community explained:

As I said, to enforce this effectively it has to be taken out of the locals' hands. It has to be like ABC, it has to be a state effort. It has to be enforced at the state level because, especially in small communities the issue is too politically charged; and when you're talking about chiefs of police that are at-will employees, it's not something that is worth risking my job for.

In one sense, the enforcers utilized their local discretion to search for pragmatic solutions to the implementation problem, while simultaneously concluding that the law would be better implemented if they had less local discretion. The state legislators had passed control to the locals, and the locals seemed to want to pass it back.

### Activists

Activists worked to generate and maintain a willingness to intervene and repair any breakdowns in the implementation process. As mentioned above, the enforcers interviewed reported that enforcement was delayed, and that many locales did nothing. Activists had a role in catalyzing enforcement. For example, one city did not enforce until it was forced to, as explained by this activist:

We worked very closely with the city attorney and actually got very involved with her office. There was talk of filing a *writ of mandamus*, and that's a very embarrassing issue for the city. Basically, we'd go into the court and say, "There's a law on the books, the city is refusing to enforce it, and we're filing a *writ of mandamus*." Now I'm not saying that we were threatening to do that ((laughter)), but let's just say that a *writ of mandamus *was discussed with the city attorney. And the city attorney, I think, looked at the situation and said, "That is a significant possibility that that could happen." I think that she was concerned it could possibly be successful, at that point contacted the mayor and chief of police, and was able to convince them that this was a significant problem of them refusing to enforce current California law. Then the mayor and chief of police put out a joint letter to all the bars and bar-restaurant combos within the city lines and started to enforce it.

The local enforcers relayed many instances when their work in bars was undermined by a lack of coordination between the various local agencies that were linked in the chain of local enforcement. Activists in non-governmental organizations (NGOs) often had an overarching perspective from which they could see where the gaps in enforcement were. Moreover, because activist organizations are typically multileveled (local, national, and international), staffers often were experienced in organizing, managing, and coordinating large-scale efforts. For example, this activist explained how their organization intervened to enhance the smooth flow of enforcement in a locality:

In (city) we had a chief of police who was strongly in support of this, but the assistant city attorney wasn't too crazy about actually prosecuting. Therefore, citations were being written, but people really weren't being prosecuted in terms that were effective. So we met with what we felt was the weak link in the chain, which was the city attorney's office. So, it depends, if you have a champion in the area and the champion is in a position of influence, then you go to that champion. If you don't have a specific champion that's in a position of influence, then often times you have to go to a weak link in the chain.

While NGOs were not in a position to make up for local government budget shortfalls, they were able to compensate by providing other resources, such as information. Organizations that had prepared for the extension of the smoking ban to bars were ready to supply bar owners with technical assistance to foster their compliance, as explained by this activist:

We have a response team of resource people. So, let's say you were a bar owner and you were going to make a patio outside, and you had never before used patio heaters. We have a person who can answer every question about patio heaters. So, what we try to do is to cover every aspect that bar owners would need to know to make their transition easier.

Furthermore, organizations gathered information on a state-wide level and distributed it to local enforcers, especially legal information. For example, *BREATH*, a project of the American Lung Association, compiled court decisions, legislative counsel, and legal opinions; provided sample warning letters and citations; and also identified resource people who could provide advice and serve as expert witnesses in local court cases. [32] Tobacco control activist organizations also tracked court challenges, as this respondent describes:

We stayed in touch through the county health departments with what was happening in the local courts. And when cases would come through, we'd speak to people who were involved and take note of what happened and then share that information with other jurisdictions. So we gathered as much information as we could about what was happening in the courts and we gave it back to the local lead agencies and we did a mailing to all the district attorneys around the state giving them an update on the court cases, gathering documents that had been written by other city attorneys and district attorneys if they tried to interpret the law.

When the case law was initially being developed, BREATH arranged a consensus conference of city attorneys to opine on what was, and was not, potentially defensible in the inevitable court challenges. All these efforts helped to fill the void that the unfunded mandate had left, especially for the local offices of the district attorneys.

## Discussion

In sum, our respondents reported that the conditions that facilitated bar owners' compliance with a smoking ban in bars included: if the cost to comply was minimal; if the bars with which they were in competition were in compliance with the smoking ban; and if there was authoritative, consistent, coordinated, and uniform enforcement. Conversely, the conditions that hindered compliance included: if the law had minimal sanctions for non-compliance; if competing bars in the area allowed smoking; and if enforcement was delayed, inadequate, or unfunded.

The lessons that could be drawn from this particular case of policy implementation have to do with giving local discretion to local implementers when dealing with a multitude of local businesses. Policy analysts who work from the bottom up tradition favor local discretion because it is a "flexible strategy that allows for adaptation to local difficulties and contextual factors."[33] However, in the case of the workplace smoking ban in bars, no amount of flexibility or adaptation was a match for the local difficulties and contextual factors.

The first local difficulty was the issue of the "level playing field." In order to protect their individual business interests, bar owners had to work collectively in unanimously complying with or unanimously ignoring the law. The slim profit margins at stake increased the focus on, and the salience of, even and consistently maintained enforcement so that no bar was at a competitive disadvantage. No amount of local discretion was going to provide local enforcers with a bigger stick or a better carrot to foster the magnitude of change required.

The second local difficulty was the very "local-ness" of enforcement. The local officials designated to enforce the smoking ban in bars did not necessarily share the state legislators' concerns regarding prophylactic measures needed to thwart a potential state workers' compensation fund catastrophe. Simply stated: the local enforcers were not motivated by the basic philosophical principles of the law. The enforcement responsibility was a new element in a typically long and ongoing relationship between local officials and local business people. The reality of local businesses' resistance to change was something that the state legislators did not have to face, and something that the local enforcers had difficulty getting beyond.

A contextual problem was the lack of implementation capacity. The state gave local enforcers the responsibility of enforcing the law, and not much else. Some of the designated local enforcers had never enforced a law before; some did not have the basic resources needed, such as time or funds. No amount of local discretion can make up for the local debits incurred by an unfunded mandate, especially to enforce a resisted law.

During the legislative hearings, public health advocates advanced the position that local discretion is good and desirable, and ultimately the best option for policy implementation. [8,9,14] This case study challenges that generalization in revealing an irony: the local people to whom local discretion was given did not want it. When their efforts to implement were thwarted, they utilized local discretion to find a solution that they had local capacity to effect: police began ticketing patrons who were smoking in the bars. Police ticketing bar patrons may be an example of using local discretion to find a new means to an end, but eventually when other implementation efforts failed, it became the end in and of itself.

Local officials who were designated as enforcers had enough experience to distinguish between policies that could be implemented locally *versus *policies that begged state implementation. The enforcers interviewed consistently proposed that the state workplace smoking ban should be implemented by the state workplace safety agency: the California Occupational Safety and Health Agency. Short of that option, experience taught them that the bar owners' most valuable and vulnerable possession was their liquor license, and including compliance with a smoking ban as a contingency in a liquor license review, as well as including a threat to a liquor license in the gradations of penalties for non-compliance with the smoking ban, would give the enforcers more leverage. However, liquor licenses were under the control of yet another state agency, the California Department of Alcoholic Beverage Control.

## Conclusion

This case study advances the implementation knowledge base in that it illuminates the dynamic of state mandates and local discretion. The implementation of the smoking ban in bars was difficult in California, yet despite the public challenges, other cities *e.g*., Mexico City,[34] counties *e.g*., Madison County, Kentucky,[2] states *e.g*., New Mexico,[2] and nations *e.g*., Uruguay,[1] have adopted the innovation of extending the workplace smoking ban to bars. Therefore, there are important lessons to be learned from this detailed empirical study of the "on the ground" operationalization of this public health policy.

In the past, government mandates that meet with local opposition, such as water fluoridation, could be implemented with simple structural solutions, such as government officials adding a substance to the water. The ease of solving that type of implementation problem is in contrast to what is needed when dealing with many small businesses that have little or no incentive to change. Implementing a smoking ban in all workplaces – including bars – requires having to designate an enforcement entity, plan coordination between various involved agencies, determine a consistent timeline for implementation, and assure resources adequate for enforcement. Our findings indicate that local implementation of a state law also requires "buy in" of the street level bureaucrats who will have to enforce it.

Furthermore, this study has implications for future research on comparable policy implementation; the policy struggle regarding worker safety from repetitive stress injury is a prescient example. Ergonomic regulations in the states of Washington and California are examples of top down policy that must be implemented in a myriad of local settings. However, in both states, the Occupational Safety and Health Agency formulated the regulation and is responsible for enforcement, thereby avoiding some of the problems inherent when one branch of government develops the policy and another is expected to enforce it.

The implementation of the workplace-smoking ban in bars demonstrates that when implementing workplace health and safety policies in the context of multiple small businesses, there are limits in the degree to which local discretion can facilitate successful policy implementation.

## Competing interests

The authors declare that they have no competing interests.

## Authors' contributions

TM conceived and designed the study, acquired the data, analyzed and interpreted the data, and drafted and revised the manuscript. LAB made substantial contributions to conception and design, or acquisition of data, or analysis and interpretation of data, and revised the manuscript critically for important intellectual content. Each author read and approved the final manuscript.

## Pre-publication history

The pre-publication history for this paper can be accessed here:



## Supplementary Material

Additional file 1 **California Labor Code Section 6404.5** Click here for file

Additional file 2 **Interview Guide for Bar Employers** Click here for file

Additional file 3 **Interview Guide for Local Enforcement Officials** Click here for file

Additional file 4 **Interview Guide for Activists** Click here for file
