# Supplementary material for: Implementation of a workplace smoking ban in bars: The limits of local discretion
Source: BMC Public Health. 2008 Dec 8;8:402. doi: 10.1186/1471-2458-8-402 (PMC2633292; doi:10.1186/1471-2458-8-402)
Supplement: Additional file 1 — California Labor Code Section 6404.5 [file 1471-2458-8-402-S1.doc]

**Appendix A: California Labor Code Section 6404.5**

1. The Legislature finds and declares that regulation of smoking in the workplace is a matter of statewide interest and concern. It is the intent of the Legislature in enacting this section to prohibit the smoking of tobacco products in all (100 percent of) enclosed places of employment in this state, as covered by this section, thereby eliminating the need of local governments to enact workplace smoking restrictions within their respective jurisdiction.

It is further the intent of the Legislature to create a uniform statewide standard to restrict and prohibit the smoking of tobacco products in enclosed places of employment, as specified in this section, in order to reduce employee exposure to environmental tobacco smoke to a level that will prevent anything other than insignificantly harmful effects to exposed employees, and also to eliminate the confusion and hardship that can result from enactment or enforcement of disparate local workplace smoking restrictions.

Notwithstanding any other provision of this section, it is the intent of the Legislature that any area not defined as a "place of employment" pursuant to subdivision (d) or in which the smoking of tobacco products is not regulated pursuant to subdivision (e) shall be subject to local regulation of smoking of tobacco products.

(b) No employer shall knowingly or intentionally permit, and no person shall engage in, the smoking of tobacco products in an enclosed space at a place of employment. "Enclosed space" includes lobbies, lounges, waiting areas, elevators, stairwells, and restrooms that are a structural part of the building and not specifically defined in subdivision (d).

(c) For purposes of this section, an employer who permits any nonemployee access to his or her place of employment on a regular basis has not acted knowingly or intentionally in violation of this section if he or she has taken the following reasonable steps to prevent smoking by a nonemployee:

1. Posted clear and prominent signs, as follows:
2. Where smoking is prohibited throughout the building or structure, a sign stating "No smoking" shall be posted at each entrance to the building or structure.
3. Where smoking is permitted in designated areas of the building or structure, a sign stating, "Smoking is prohibited except in designated areas" shall be posted at each entrance to the building or structure.
4. Has requested, when appropriate, that a nonemployee who is smoking refrain from smoking in the enclosed workplace.

For purposes of this subdivision, "reasonable steps" does not include (A) the physical ejection of a nonemployee from the place of employment or (B) any requirement for making a request to a nonemployee to refrain from smoking, under circumstances involving a risk of physical harm to the employer or any employee.

(d) For purposes of this section, "place of employment" does not include any of the following:

1. Sixty-five percent of the guestroom accommodations in a hotel, motel, or similar transient lodging establishment.
2. Areas of the lobby in a hotel, motel, or other similar transient lodging establishment designated for smoking by the establishment. An establishment may permit smoking in a designated lobby area that does not exceed 25 percent of the total floor area of the lobby or, if the total area of the lobby is 2,000 square feet or less, that does not exceed 50 percent of the total floor area of the lobby. For purposes of this paragraph, "lobby" means the common public area of an establishment in which registration and other similar or related transactions, or both, are conducted and in which the establishment's guests and members of the public typically congregate.
3. Meeting and banquet rooms in a hotel, motel, other transient lodging establishment similar to a hotel or motel, restaurant, or public convention center, except while food or beverage functions are taking place, including setup, service, and cleanup activities, or when the room is being used for exhibit purposes. At times when smoking is not permitted in a meeting or banquet room pursuant to this paragraph, the establishment may permit smoking in corridors and pre-function areas adjacent to and serving the meeting or banquet room if no employee is stationed in that corridor or area on other than a passing basis.
4. Retail or wholesale tobacco shops and private smokers' lounges. For purposes of this paragraph:
5. "Private smokers' lounge" means any enclosed area in or attached to a retail or wholesale tobacco shop that is dedicated to the use of tobacco products, including, but not limited to, cigars and pipes.
6. "Retail or wholesale tobacco shop" means any business establishment the main purpose of which is the sale of tobacco products, including, but not limited to, cigars, pipe tobacco, and smoking accessories.
7. Cabs of motor trucks, as defined in Section 410 of the Vehicle Code, or truck tractors, as defined in Section 655 of the Vehicle Code, if no nonsmoking employees are present.
8. Warehouse facilities. For purposes of this paragraph, "warehouse facility" means a warehouse facility with more than 100,000 square feet of total floor space, and 20 or fewer full-time employees working at the facility, but does not include any area within a facility that is utilized as office space.
9. Gaming clubs, in which smoking is permitted by subdivision (f). For purposes of this paragraph, "gaming club" means any gaming club, as defined in Section 19802 of the Business and Professions Code, or bingo facility, as defined in Section 326.5 of the Penal Code, that restricts access to minors under 18 years of age.
10. Bars and taverns, in which smoking is permitted by subdivision (f). For purposes of this paragraph, "bar" or "tavern" means a facility primarily devoted to the serving of alcoholic beverages for consumption by guests on the premises, in which the serving of food is incidental. "Bar or tavern" includes those facilities located within a hotel, motel, or other similar transient occupancy establishment. However, when located within a building in conjunction with another use, including a restaurant, "bar" or "tavern" includes only those areas used primarily for the sale and service of alcoholic beverages. "Bar" or "tavern" does not include the dining areas of a restaurant, regardless of whether alcoholic beverages are served therein.
11. Theatrical production sites, if smoking is an integral part of the story in the theatrical production.
12. Medical research or treatment sites, if smoking is integral to the research and treatment being conducted.
13. Private residences, except for private residences licensed as family day care homes, during the hours of operation as family day care homes and in those areas where children are present.
14. Patient smoking areas in long-term health care facilities, as defined in Section 1418 of the Health and Safety Code.
15. Break rooms designated by employers for smoking, provided that all of the following conditions are met:
16. Air from the smoking room shall be exhausted directly to the outside by an exhaust fan. Air from the smoking room shall not be re-circulated to other parts of the building.
17. The employer shall comply with any ventilation standard or other standard utilizing appropriate technology, including, but not limited to, mechanical, electronic, and biotechnical systems, adopted by the Occupational Safety and Health Standards Board or the federal Environmental Protection Agency. If both adopt inconsistent standards, the ventilation standards of the Occupational Safety and Health Standards Board shall be no less stringent than the standards adopted by the federal Environmental Protection Agency.
18. The smoking room shall be located in a non-work area where no one, as part of his or her work responsibilities, is required to enter. For purposes of this subparagraph, "work responsibilities" does not include any custodial or maintenance work carried out in the break room when it is unoccupied.
19. There are sufficient nonsmoking break rooms to accommodate nonsmokers.
20. Employers with a total of five or fewer employees, either full time or part time, may permit smoking where all of the following conditions are met:
21. The smoking area is not accessible to minors.
22. All employees who enter the smoking area consent to permit smoking. No one, as part of his or her work responsibilities, shall be required to work in an area where smoking is permitted. An employer who is determined by the division to have used coercion to obtain consent or who has required an employee to work in the smoking area shall be subject to the penalty provisions of Section 6427.
23. Air from the smoking area shall be exhausted directly to the outside by an exhaust fan. Air from the smoking area shall not be re-circulated to other parts of the building.
24. The employer shall comply with any ventilation standard or other standard utilizing appropriate technology, including, but not limited to, mechanical, electronic, and biotechnical systems, adopted by the Occupational Safety and Health Standards Board or the federal Environmental Protection Agency. If both adopt inconsistent standards, the ventilation standards of the Occupational Safety and Health Standards Board shall be no less stringent than the standards adopted by the federal Environmental Protection Agency.

This paragraph shall not be construed to (i) supersede or render inapplicable any condition or limitation on smoking areas made applicable to specific types of business establishments by any other paragraph of this subdivision or (ii) apply in lieu of any otherwise applicable paragraph of this subdivision that has become inoperative.

(e) Paragraphs (13) and (14) of subdivision (d) shall not be construed to require employers to provide reasonable accommodation to smokers, or to provide break rooms for smokers or nonsmokers.

(f) (1) Except as otherwise provided in this subdivision, smoking may be permitted in gaming clubs, as defined in paragraph (7) of subdivision (d), and in bars and taverns, as defined in paragraph (8) of subdivision (d), until the earlier of the following:

1. January 1, 1998.
2. The date of adoption of a regulation (i) by the Occupational Safety and Health Standards Board reducing the permissible employee exposure level to environmental tobacco smoke to a level that will prevent anything other than insignificantly harmful effects to exposed employees or (ii) by the federal Environmental Protection Agency establishing a standard for reduction of permissible exposure to environmental tobacco smoke to an exposure level that will prevent anything other than insignificantly harmful effects to exposed persons.
3. If a regulation specified in subparagraph (B) of paragraph (1) is adopted on or before January 1, 1998, smoking may thereafter be permitted in gaming clubs and in bars and taverns, subject to full compliance with, or conformity to, the standard in the regulation within two years following the date of adoption of the regulation. An employer failing to achieve compliance with, or conformity to, the regulation within this two-year period shall prohibit smoking in the gaming club, bar, or tavern until compliance or conformity is achieved. If the Occupational Safety and Health Standards Board and the federal Environmental Protection Agency both adopt regulations specified in subparagraph (B) of paragraph (1) that are inconsistent, the regulations of the Occupational Safety and Health Standards Board shall be no less stringent than the regulations of the federal Environmental Protection Agency.
4. If a regulation specified in subparagraph (B) of paragraph (1) is not adopted on or before January 1, 1998, the exemptions specified in paragraphs (7) and (8) of subdivision (d) shall become inoperative on and after January 1, 1998, until a regulation is adopted. Upon adoption of such a regulation on or after January 1, 1998, smoking may thereafter be permitted in gaming clubs and in bars and taverns, subject to full compliance with, or conformity to, the standard in the regulation within two years following the date of adoption of the regulation. An employer failing to achieve compliance with, or conformity to, the regulation within this two-year period shall prohibit smoking in the gaming club, bar, or tavern until compliance or conformity is achieved. If the Occupational Safety and Health Standards Board and the federal Environmental Protection Agency both adopt regulations specified in subparagraph (B) of paragraph (1) that are inconsistent, the regulations of the Occupational Safety and Health Standards Board shall be no less stringent than the regulations of the federal Environmental Protection Agency.
5. From January 1, 1997, to December 31, 1997, inclusive, smoking may be permitted in gaming clubs, as defined in paragraph (7) of subdivision (d), and in bars and taverns, as defined in paragraph (8) of subdivision (d), subject to both of the following conditions:
6. If practicable, the gaming club or bar or tavern shall establish a designated nonsmoking area.
7. If feasible, no employee shall be required, in the performance of ordinary work responsibilities, to enter any area in which smoking is permitted.

(g) The smoking prohibition set forth in this section shall constitute a uniform statewide standard for regulating the smoking of tobacco products in enclosed places of employment and shall supersede and render unnecessary the local enactment or enforcement of local ordinances regulating the smoking of tobacco products in enclosed places of employment. Insofar as the smoking prohibition set forth in this section is applicable to all (100-percent) places of employment within this state and, therefore, provides the maximum degree of coverage, the practical effect of this section is to eliminate the need of local governments to enact enclosed workplace smoking restrictions within their respective jurisdictions.

(h) Nothing in this section shall prohibit an employer from prohibiting smoking in an enclosed place of employment for any reason.

(i) The enactment of local regulation of smoking of tobacco products in enclosed places of employment by local governments shall be suspended only for as long as, and to the extent that, the (100-percent) smoking prohibition provided for in this section remains in effect. In the event this section is repealed or modified by subsequent legislative or judicial action so that the (100-percent) smoking prohibition is no longer applicable to all enclosed places of employment in California, local governments shall have the full right and authority to enforce previously enacted, and to enact and enforce new, restrictions on the smoking of tobacco products in enclosed places of employment within their jurisdictions, including a complete prohibition of smoking. Notwithstanding any other provision of this section, any area not defined as a "place of employment" or in which smoking is not regulated pursuant to subdivision (d) or (e), shall be subject to local regulation of smoking of tobacco products.

(j) Any violation of the prohibition set forth in subdivision (b) is an infraction, punishable by a fine not to exceed one hundred dollars ($100) for a first violation, two hundred dollars ($200) for a second violation within one year, and five hundred dollars ($500) for a third and for each subsequent violation within one year. This subdivision shall be enforced by local law enforcement agencies, including, but not limited to, local health departments, as determined by the local governing body.

(k) Notwithstanding Section 6309, the division shall not be required to respond to any complaint regarding the smoking of tobacco products in an enclosed space at a place of employment, unless the employer has been found guilty pursuant to subdivision (j) of a third violation of subdivision (b) within the previous year.

(l) If any provision of this act or the application thereof to any person or circumstances is held invalid, that invalidity shall not affect other provisions or applications of the act that can be given effect without the invalid provision or application, and to this end the provisions of this act are severable.
