# Supplementary material for: Implementation of a workplace smoking ban in bars: The limits of local discretion
Source: BMC Public Health. 2008 Dec 8;8:402. doi: 10.1186/1471-2458-8-402 (PMC2633292; doi:10.1186/1471-2458-8-402)
Supplement: Additional file 2 — Interview Guide for Bar Employers [file 1471-2458-8-402-S2.doc]

**Appendix B: Interview Guide for Bar Employers**

Describe your experiences with the implementation of the smoking ban that took effect January 1, 1998.

What previous experiences did you have with smoking bans (such as local ordinances)?

Have you developed any ways to allow patrons or workers to continue to smoke in your bar?

What you have done, or what would you do when a patron or worker lights a cigarette in your bar?

How about your workers (bartenders/servers) -- what are they doing if they need to have a cigarette while working?

Describe any enforcement experiences.

Tell me about your experiences with enforcement officials.

What local agency is responsible for enforcing the law in your area? Have you had contact with anyone enforcing the law?

Have there been any court cases in your area with respect to the smoking ban in bars?

What other experiences have you had with local enforcement of laws, codes, or regulations?

Have any groups contacted you or worked with you on this issue?

(*e.g.*, labor unions, Cal OSHA, Americans for Nonsmokers' Rights, BREATH, California State Department of Health, or local government agents)

(*e.g.*, California Licensed Food and Beverage Association, The Northern California Tavern and Restaurant Association, F.O.R.C.E.S., the National Smokers' Alliance)

Please **profile** your bar for me in terms of:

• the type of area in which the bar is located (*e.g*., urban, suburban, subrural, rural), (*e.g.,* commercial, residential, tourist, manufacturing, retail);

• the type of patrons the bar caters to (e.g., tourists, locals, party, and commuters);

• the type of owner of the premises (*e.g*., national chain, small business, owner-operated).

What other comparable bars in the area would you consider your direct competitors with respect to your clientele? (*e.g*., many comparable bars, a few comparable bars, no other bars in the area)
