# Supplementary material for: Implementation of a workplace smoking ban in bars: The limits of local discretion
Source: BMC Public Health. 2008 Dec 8;8:402. doi: 10.1186/1471-2458-8-402 (PMC2633292; doi:10.1186/1471-2458-8-402)
Supplement: Additional file 3 — Interview Guide for Local Enforcement Officials [file 1471-2458-8-402-S3.doc]

**Appendix C: Interview Guide for Local Enforcement Officials**

Tell me about the local political process that led to your agency becoming the enforcer of Labor Code 6404.5.

What has been happening concerning enforcement since AB 13 went into effect in January 1995?

How has your agency been involved in the extension of AB 13 to cover bar employees in January 1, 1998?

What other agencies have been involved? (*e.g.,* county health department, local tobacco control programs, local government officials, voluntary health organizations (*i.e.,* ACS, ALA), education (schools), Businesses (restaurant associations), anti-smoking coalitions (*i.e.,* ASH/ANR)

What types of enforcement activities have you done with respect to Labor Code 6404.5? (*e.g.,* responded to inquiries, responded to complaints, issued warnings, issued citations, issued fines, conducted compliance checks, educated individuals and groups)

Have you developed any overarching strategies for enforcement?

Are you aware of any strategies that bar employers in your area have developed to circumvent the smoking ban in bars to allow patrons or workers to continue to smoke in their bar?

Have there been any court cases in your area, where bar owners or patrons challenged your enforcement? What is your relationship with the District Attorney in your area?

What is your assessment of your agency's effectiveness in enforcing Labor Code 6404.5? In the hospitality industry in general? In banning smoking in bars in particular?

What conditions facilitate effective enforcement?

What conditions hinder enforcement?

What other codes, laws, or regulations are you responsible for enforcing? How is the enforcement of Labor Code 6404.5 similar or different from enforcing other laws, codes, or regulations?

In your particular area, who have been the biggest supporters of the smoking ban in bars?

In your particular area, who have been the biggest opponents of the smoking ban in bars?
