# Supplementary material for: Implementation of a workplace smoking ban in bars: The limits of local discretion
Source: BMC Public Health. 2008 Dec 8;8:402. doi: 10.1186/1471-2458-8-402 (PMC2633292; doi:10.1186/1471-2458-8-402)
Supplement: Additional file 4 — Interview Guide for Activists [file 1471-2458-8-402-S4.doc]

**Appendix D: Interview Guide for Activists**

What has been the nature and extent of your agency/organization's involvement in support of or in opposition to AB 13?

More specifically, I would like to know when, how, and why your agency/organization got involved, and how your activism has changed over time.

What types of activism did you engage in, either to support or oppose AB 13? (Probes: organize voters, petition, lobby legislators, protests, rallies, marches, demonstrations, testify at hearings, boycott or picket, research, voter registration, press conference, lobby the DA, *etc*.)

What other businesses/agencies/organizations did you collaborate with in your efforts?

What types of media coverage did your efforts engender?

What types of activities did your agency/organization engage in with respect to the recent extension of the Labor Code 6404.5 smoking ban in bars on January 1, 1998?

How do these efforts compare to other advocacy in which you've been involved?

What is your assessment of Labor Code 6404.5's effectiveness in curtailing smoking in the hospitality industry in general?

What is your assessment of Labor Code 6404.5's effectiveness in curtailing smoking in bars in particular?

What is a good target effectiveness for the workplace in general; for bars in particular?
